# Association Between Autism Spectrum Disorders With or Without Intellectual Disability and Depression in Young Adulthood

**DOI:** 10.1001/jamanetworkopen.2018.1465

**Published:** 2018-08-31

**Authors:** Dheeraj Rai, Hein Heuvelman, Christina Dalman, Iryna Culpin, Michael Lundberg, Peter Carpenter, Cecilia Magnusson

**Affiliations:** 1 National Institute for Health Research Biomedical Research Centre, University of Bristol, Bristol, United Kingdom; 2 Department of Public Health Sciences, Karolinska Institutet, Stockholm, Sweden; 3 Centre for Academic Mental Health, Population Health Sciences, Bristol Medical School, University of Bristol, Bristol, United Kingdom; 4 Avon and Wiltshire Partnership National Health Service Mental Health Trust, Bristol, United Kingdom; 5 Centre for Epidemiology and Community Medicine, Stockholm Health Care Services, Stockholm, Sweden

## Abstract

**Importance:**

Depression is a frequently occurring mental disorder and may be common in adults with autism spectrum disorders (ASD), but there is a lack of longitudinal population-based studies examining this association. Whether any increased risk of depression in ASD has a shared familial basis and whether it differs by co-occurring intellectual disability is not well known.

**Objectives:**

To examine whether individuals with ASD are more likely to be diagnosed as having depression in adulthood than the general population and their nonautistic siblings and to investigate whether these risks differ by the presence or absence of intellectual disability.

**Design, Setting, and Participants:**

Population-based cohort study with a nested sibling comparison. The Stockholm Youth Cohort is a total population record linkage study that includes all children and young people (age range, 0-17 years) who were ever resident in Stockholm County, Sweden, between January 2001 and December 2011 (n = 735 096). Data analysis was conducted between January 5 and November 30, 2017, in Stockholm County, Sweden.

**Main Outcomes and Measures:**

Clinical diagnosis of depressive disorders was identified using the Stockholm County Adult Psychiatric Outpatient Register and the Swedish National Patient Register.

**Results:**

Participants were 223 842 individuals followed up to age 27 years by 2011, of whom 4073 had diagnosed ASD (mean [SD] age, 21.5 [2.7] years; 65.9% male; 2927 without intellectual disability and 1146 with intellectual disability) and 219 769 had no ASD (mean [SD] age, 22.1 [2.8] years; 50.9% male). By age 27 years, 19.8% (n = 808) of individuals diagnosed having ASD had a diagnosis of depression compared with 6.0% (n = 13 114) of the general population (adjusted relative risk [RR], 3.64; 95% CI, 3.41-3.88). The risk of a depression diagnosis was higher in ASD without intellectual disability (adjusted RR, 4.28; 95% CI, 4.00-4.58) than in ASD with intellectual disability (adjusted RR, 1.81; 95% CI, 1.51-2.17). Nonautistic full-siblings (adjusted RR, 1.37; 95% CI, 1.23-1.53) and half-siblings (adjusted RR, 1.42; 95% CI, 1.23-1.64) of individuals with ASD also had a higher risk of depression than the general population. Compared with their nonautistic full-siblings, individuals with ASD had more than a 2-fold risk of a depression diagnosis (adjusted odds ratio, 2.50; 95% CI, 1.91-3.27) in young adulthood.

**Conclusions and Relevance:**

According to this study’s results, ASD, particularly ASD without intellectual disability, is associated with depression by young adulthood compared with the general population. It appears that this association is unlikely to be explained by shared familial liability. Future research to identify modifiable pathways between ASD and depression may assist in the development of preventive interventions.

## Key Points

**Questions:**

Are individuals with autism spectrum disorders more likely to have depression in adulthood than the general population, and do these risks have a familial basis and differ by coexisting intellectual disability?

**Findings:**

In this Swedish population-based cohort study of 223 842 participants with a nested sibling comparison, individuals with autism spectrum disorders, especially those without intellectual disability, had a greater risk of a depression diagnosis in young adulthood than the general population and their nonautistic siblings.

**Meaning:**

According to this study’s results, depression is overrepresented in autism spectrum disorders, and this higher risk may not be explained by shared familial liability; research identifying modifiable pathways may help develop preventive interventions.

## Teaser

This population-based cohort study with a nested sibling comparison examines whether Swedish individuals with autism spectrum disorders are more likely to be diagnosed as having depression in adulthood than the general population and their nonautistic siblings and investigates whether these risks differ by the presence or absence of intellectual disability.

## Introduction

Autism spectrum disorders (ASD) are developmental conditions with difficulties in reciprocal social interaction and restricted and repetitive behaviors and interests.^1^ Despite the dramatic rise in the recognition of ASD in recent decades,^2,3,4^ little is known about the outcomes or service needs of people with ASD in adulthood.^5^

Mental health problems, such as depression, are considered to be common in individuals with ASD.^6,7,8^ However, most studies to date have been conducted in pediatric populations, with findings difficult to extrapolate to adulthood,^9^ especially considering some studies report that depressive features improve with age in children with ASD.^10,11^ Previous studies among adults with ASD have largely been cross-sectional and carried out in selected clinical populations without comparison groups, reporting a wide range of estimates of the prevalence of depression.^8,12,13,14,15^ To our knowledge, the only large population-based study with a comparison group included 1507 adults diagnosed as having ASD and reported a 25% prevalence of depression, representing a 2-fold increased risk of depression in ASD.^15^ That study did not differentiate autism by co-occurring intellectual disability, which is important considering the suggestion that individuals with ASD who have greater cognitive abilities may be particularly prone to depression because of greater awareness of their difficulties.^9,16,17,18^

Furthermore, the mechanisms behind any increased risk of depression in people with ASD are not well understood. The possibility of a common genetic architecture between different psychiatric conditions has been highlighted,^19^ and a shared genetic vulnerability between autism and psychiatric comorbidities is plausible. Autism and depression are both heritable conditions, and a higher prevalence of affective disorders among parents, particularly mothers, as well as siblings of individuals with autism, has been reported.^20,21^ However, large studies^19,22^ by the Cross-Disorder Group of the Psychiatric Genomics Consortium have not found evidence to support any strong shared genetic overlap between autism and depression. Estimating the risks in siblings can assist in the understanding of any possible familial basis of these associations, and sibling comparisons can be a powerful method to account for shared familial factors (genetic and environmental) and hence address the problem of confounding that is common in observational studies.

Improving our understanding of the burden of depression in people with autism is important because it is known to lead to additional disability and reduced social functioning. Depression may also exacerbate the core features of ASD^16^ and is a strong risk factor for suicide,^23^ which has recently been highlighted as a potentially important cause of premature mortality in higher-functioning individuals with autism.^24^ Because depression is potentially treatable, its identification and management in people with ASD may thus offer the opportunity to reduce distress and lead to an improved quality of life.

We investigated the Stockholm Youth Cohort, a large total population record linkage study in Stockholm County, Sweden. Our main aims were to (1) examine whether individuals with ASD are more likely to be diagnosed as having depression in adulthood than the general population and their nonautistic siblings and (2) investigate whether these risks differ by the presence or absence of intellectual disability.

## Methods

### Study Cohort

The Stockholm Youth Cohort includes all children and young people (age range, 0-17 years) who were ever resident in Stockholm County, Sweden, between January 1, 2001, and December 31, 2011 (n = 735 096).^25^ A vast range of Swedish national and regional registries has been used to collect data on cohort members and their first-degree and second-degree relatives. The most recent update of the cohort includes data until December 31, 2011, when the oldest cohort members were age 27 years. Ethical approval for the record linkages without individual informed consent and the use of anonymized data for the study was obtained from the Research Ethics Committee at Karolinska Institutet, Stockholm, Sweden. Data analysis was conducted between January 5 and November 30, 2017. We used the Strengthening the Reporting of Observational Studies in Epidemiology (STROBE) reporting guidelines for observational studies to guide the reporting of this study.

Because our primary outcome was a diagnosis of depression in adulthood, the eligible cohort included individuals followed up until they were at least 18 years old (n = 223 842) (Figure) by December 31, 2011. We identified the full-siblings (those who had the same biological parents as the index person) and half-siblings (those with 1 common biological parent) of these individuals using the Swedish Multigenerational Register.

**Figure.**
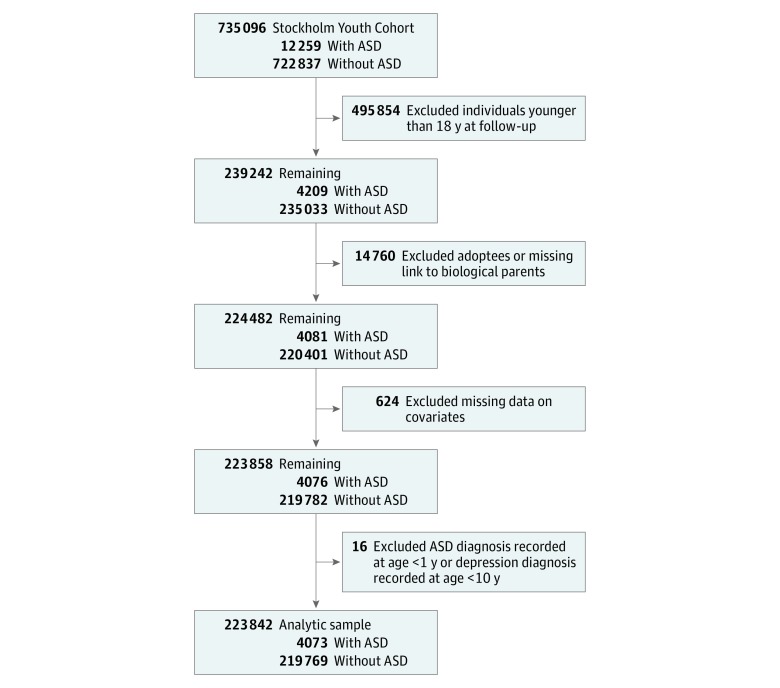
Flowchart of Selection Procedure for the Study Population From the Stockholm Youth Cohort ASD indicates autism spectrum disorders.

### Ascertainment of ASD

Autism spectrum disorders have been identified in the Stockholm Youth Cohort using record linkage with registries covering all known publicly funded pathways of assessment, care, or special education for ASD in Stockholm County.^25^ Using this multisource ascertainment method, 12 259 individuals (1.7%) within the cohort have been identified as having a diagnosis of ASD.^3^ A record of co-occurring intellectual disability in individuals with ASD was identified using the relevant codes of the *International Classification of Diseases*, *Ninth Revision* (*ICD-9*) (codes 317-319), *International Statistical Classification of Diseases and Related Health Problems, Tenth Revision *(*ICD-10*) (codes F70-79), and *Diagnostic and Statistical Manual of Mental Disorders*, *Fourth Edition* (*DSM-IV*) (codes 317-319) in the child and adult mental health registers or the Swedish National Patient Register. Two validation studies have previously been carried out, including a case note review by clinical experts and a cross-validation study with a national study of twins, which both highlighted the validity of the cases recorded as having autism in the Stockholm Youth Cohort.^25^ Considering that rates of depression may vary by the level of cognitive ability, as well as previous work highlighting differences in the antecedents^26,27^ and outcomes^28^ of ASD by intellectual disability, apart from studying ASD as a combined group, we also dichotomized individuals with ASD based on the presence or absence of intellectual disability recorded in the registers.^25^

### Ascertainment of Depression

We used 2 sources to identify a clinically recorded diagnosis of depressive disorders (*ICD-10* codes F32-39) from age 18 years up to a maximum age of 27 years. These included the Stockholm County Adult Psychiatric Outpatient Register, which records the dates and diagnoses for any contact since 1997 with specialist outpatient psychiatric services in Stockholm County,^29^ and the Swedish National Patient Register, which contains the dates and discharge diagnoses of all inpatients since 1973, as well as specialist outpatients since 2001 (although with incomplete psychiatric outpatient data) in Sweden. Depression diagnoses in Swedish registers have been shown to have a fair to moderate agreement against a clinical registry with standardized diagnoses (κ = 0.32; 88% full agreement),^30^ and twins with a depression diagnosis in the national patient registers were reported to have substantially higher depressive symptom scores than those without recorded depression.^30^ We also extracted data on these diagnoses before age 18 years from the Swedish National Patient Register and the Stockholm County Child and Adolescent Mental Health Register^25^ for sensitivity analysis as described below in the Statistical Analysis subsection.

### Potential Confounders

A number of variables were considered as potential confounders. These included age and sex of the child, maternal and paternal age,^31^ highest educational level of either parent (≤9, 10-12, or ≥13 years), disposable family income adjusted for year of ascertainment and family size (in quintiles),^32^ migrant household (defined as a parent or the child born outside of Sweden),^26^ and any history of a psychiatric disorder in the mother or the father.^27^

### Statistical Analysis

After descriptive analysis of the distribution of outcomes and covariates in the comparison groups, we assessed the association between ASD (and ASD without or with intellectual disability) and depression using Poisson regression models with robust error variance as described by Zou.^33^ First, we calculated relative risks (RRs) and used robust SEs to derive 95% CIs, accounting for clustering of children born to the same mother. The initial models were adjusted for age and sex (model 1) followed by additional adjustment for maternal and paternal age, highest educational level of the mother or father, disposable family income adjusted for family size at birth in quintiles, birth of parents or child outside of Sweden, and a record of any psychiatric disorders in the mother or father (model 2).

Second, we assessed the risk of depression in full-siblings and half-siblings of individuals with ASD compared with the general population. Because full-siblings share 50% of their cosegregating genes, they are genetically more similar than half-siblings, who share 25% of their cosegregating genes. Therefore, if the association between autism and depression was primarily of genetic origin, we would expect the RR of depression in autism to be highest, followed by nonautistic full-siblings and half-siblings of people with autism.

Third, to directly compare the risk of depression in individuals with ASD with that of their nonautistic siblings, we matched ASD cases with up to 2 nonautistic full-siblings of the same sex. To account for the matched case-control design, we carried out fixed-effects conditional logistic regression analysis to estimate the odds of depression in individuals with ASD vs that in their nonautistic full-siblings. For these analyses, we adjusted for age and birth order (model 1) and additionally for maternal and paternal age at birth (model 2) because these may represent nonshared characteristics between sibling pairs.

Because a diagnosis of depression may precede a diagnosis of ASD, especially in individuals without an intellectual disability,^12^ we repeated our main analysis after removing individuals who received a diagnosis of ASD following a recorded depression diagnosis. In another sensitivity analysis, we estimated the associations between ASD and adult depression, while adjusting for any depression diagnoses received in child and adolescent mental health services before age 18 years. All analyses were conducted using statistical software (Stata, version 14; StataCorp LP). Two-sided *P* < .05 indicated statistical significance.

## Results

For our main analyses, we had 223 842 individuals born to 144 558 mothers followed up in the registers from at least age 18 years up to age 27 years by 2011. Of these, 4073 had a diagnosis of ASD (mean [SD] age, 21.5 [2.7] years; 2673 [65.9%] male) (2927 without intellectual disability and 1146 with intellectual disability), and 219 769 had no diagnosis of ASD (mean [SD] age, 22.1 [2.8] years; 111 794 [50.9%] male). Between the ages of 18 and 27 years, 808 (19.8%) of the individuals with ASD had received a diagnosis of depression compared with 13 114 (6.0%) of the population without a diagnosis of ASD (Table 1). Depression appeared to be more prevalent among people with ASD without intellectual disability (704 [24.1%]) than among those with ASD with intellectual disability (104 [9.1%]). This corresponded to an adjusted RR of 3.64 (95% CI, 3.41-3.88), which was greater for ASD without intellectual disability (adjusted RR, 4.28; 95% CI, 4.00-4.58) and was also increased for ASD with intellectual disability (adjusted RR, 1.81; 95% CI, 1.51-2.17) (Table 2).

**Table 1.** Characteristics of the Eligible Study Population Within the Stockholm Youth Cohort by ASD Without and With ID

Variable	No ASD (n = 219 769)	All ASD (n = 4073)	ASD Without ID (n = 2927)	ASD With ID (n = 1146)
Age, mean (SD), y	22.1 (2.8)	21.5 (2.7)	21.4 (2.6)	21.5 (2.8)
Male, No. (%)	111 794 (50.9)	2683 (65.9)	1882 (64.3)	801 (69.9)
Born to nulliparous mother, No./total No. (%)	87 702/198 616 (44.2)	1814/3871 (46.9)	1356/2810 (48.3)	458/1061 (43.2)
Age at birth, mean (SD), y				
Mother	28.6 (5.3)	29.2 (5.6)	29.0 (5.6)	29.5 (5.7)
Father	31.7 (6.3)	32.1 (6.7)	31.8 (6.7)	32.7 (6.8)
Low educational level of parents, No. (%)^a^	71 279 (32.4)	1323 (32.5)	912 (31.2)	411 (35.9)
Lowest quintile of family income, No. (%)	49 803 (22.7)	795 (19.5)	516 (17.6)	279 (24.3)
Individual or parents born outside of Sweden, No. (%)	77 644 (35.3)	1276 (31.3)	827 (28.3)	449 (39.2)
Psychiatric history, No. (%)				
Mother	68 536 (31.2)	1854 (45.5)	1405 (48.0)	449 (39.2)
Father	41 287 (18.8)	1071 (26.3)	794 (27.1)	277 (24.2)
Individual has psychiatric comorbidities other than depression, No. (%)	42 676 (19.4)	2825 (69.4)	1923 (65.7)	902 (78.7)
Adult depression at follow-up, No. (%)	13 114 (6.0)	808 (19.8)	704 (24.1)	104 (9.1)

Abbreviations: ASD, autism spectrum disorders; ID, intellectual disability.

^a^ One or both parents completed no more than compulsory 9-year schooling.

**Table 2.** Relative Risk of Adult Depression Among Those Diagnosed as Having ASD and Among Their Full-Siblings and Half-Siblings Compared With Population Controls^a^

Variable	RR (95% CI)^b^					
	Cases vs Population Controls		Full-Siblings of Cases vs Population Controls		Half-Siblings of Cases vs Population Controls	
	Model 1	Model 2	Model 1	Model 2	Model 1	Model 2
All ASD	4.19 (3.94-4.47)	3.64 (3.41-3.88)	1.52 (1.36-1.69)	1.37 (1.23-1.53)	1.73 (1.51-2.00)	1.42 (1.23-1.64)
*P* value	<.001	<.001	<.001	<.001	<.001	<.001
ASD without ID	5.05 (4.73-5.39)	4.28 (4.00-4.58)	1.69 (1.50-1.91)	1.47 (1.31-1.66)	1.78 (1.52-2.07)	1.45 (1.24-1.69)
*P* value	<.001	<.001	<.001	<.001	<.001	<.001
ASD with ID	1.96 (1.63-2.35)	1.81 (1.51-2.17)	1.09 (0.87-1.38)	1.08 (0.86-1.36)	1.58 (1.15-2.17)	1.33 (0.98-1.81)
*P* value	<.001	<.001	.45	.49	.005	.07

Abbreviations: ASD, autism spectrum disorders; ID, intellectual disability; RR, relative risk.

^a^ By modified Poisson regression with cluster robust SEs.

^b^ Model 1 adjusted for age and sex. Model 2 adjusted for age, sex, maternal and paternal age, highest educational level of either parent, disposable family income quintile, migrant household, and any history of a psychiatric disorder in the mother or the father.

The corresponding risk estimates for depression in the full-siblings and half-siblings of cohort members with autism, who themselves had never received a diagnosis of ASD, are listed in Table 2. Nonautistic full-siblings (adjusted RR, 1.37; 95% CI, 1.23-1.53) and half-siblings (adjusted RR, 1.42; 95% CI, 1.23-1.64) of individuals with ASD also had a higher risk of depression compared with population controls, but no clear risk gradient was observed between these groups. These associations were more apparent for siblings of children with ASD without intellectual disability.

Finally, we directly compared individuals with ASD with up to 2 sex-matched nonautistic siblings in relation to their risk of depression. Compared with their nonautistic full-siblings, individuals with ASD had more than a 2-fold risk of a depression diagnosis (adjusted odds ratio, 2.50; 95% CI, 1.91-3.27) in young adulthood, and this association also seemed largely driven by an overrepresentation of depression in ASD without intellectual disability (Table 3).

**Table 3.** Odds Ratios for Adult Depression Among Autism Spectrum Disorder Cases Compared With Sex-Matched Sibling Controls^a^

Variable	OR (95% CI)		No. of Adult Depression Diagnoses/Total No. at Risk	
	Model 1^b^	Model 2^c^	ASD Cases	Sibling Controls
All ASD	2.44 (1.87-3.19)	2.50 (1.91-3.27)	199/1188	117/1353
ASD without ID	3.39 (2.46-4.69)	3.49 (2.52-4.84)	172/785	80/890
ASD with ID	1.00 (0.57-1.75)	0.97 (0.55-1.72)	25/368	32/422

Abbreviations: ASD, autism spectrum disorders; ID, intellectual disability; OR, odds ratio.

^a^ By conditional logistic regression.

^b^ Adjusted for age and birth order.

^c^ Adjusted for age, birth order, and maternal and paternal age.

Approximately half (n = 483) of individuals with ASD who had a diagnosis of depression (equating to 11.9% of the 4073 individuals with ASD) in our study received a diagnosis of ASD only after they had already been diagnosed as having depression. When we removed these individuals and repeated the analysis, the strong associations between ASD and depression persisted, although with some attenuation of the estimates, particularly for the ASD without intellectual disability group (eTable 1 in the Supplement). The associations also persisted in a further sensitivity analysis in which we adjusted our main analysis for depression diagnoses received in child and adolescent mental health services before age 18 years (eTable 2 and eTable 3 in the Supplement).

## Discussion

In this population-based cohort study with a nested sibling comparison in Sweden, individuals with ASD had a substantially greater risk of a depression diagnosis in young adulthood than the general population. This risk increase was more pronounced than that in their nonautistic siblings, who themselves had a higher risk of depression than the general population. The associations with depression were stronger in individuals with autism who did not have intellectual disability.

Our findings concur with evidence suggesting a high burden of depression in adults with ASD,^8^ although longitudinal studies with a comparison group have been, to our knowledge, absent from the literature. That we know of, there is only 1 previous population-based study^15^ with which our results can be directly compared. That study used insurance claims data in California and reported that more than one-quarter of 1507 individuals with autism also had a recorded diagnosis of depression, equating to greater than a 2-fold increased odds of depression.^15^ The prevalence of depression among our sample of more than 4000 individuals with ASD is somewhat lower, probably reflecting differences in diagnostic practice between Sweden and the United States. To our knowledge, no previous population-based study has highlighted the stark differences in risk of depression among individuals with ASD by the presence or absence of intellectual disability or has used sibling comparisons to demonstrate increased risk of depression among individuals with ASD over and beyond any influence of shared genetics and environmental factors. Our results are consistent with a recent study^21^ that reported a greater burden of affective disorders in siblings of individuals with ASD compared with siblings of nonautistic control children, although it did not report on rates of depression in individuals with ASD.

The data linkages allowed us to estimate the rates of depression diagnoses in nonautistic full-siblings and half-siblings of our ASD cases. Such designs can help identify gradients in risk that may be influenced by genetic loading. Our results did not suggest any risk gradients between full-siblings and half-siblings, although both of these groups appeared to have a greater risk of depression than the general population. While the increased risk of depression in siblings may still suggest the role of common genetic vulnerability, siblings of children with ASD may also be prone to psychiatric disorders through other mechanisms. For example, they may receive less parental attention because of their autistic sibling with greater needs or may experience other stressors or discord in the household, or they may be more likely to have closer monitoring because their sibling has a diagnosis of autism. We also directly compared the risks of depression in ASD cases vs matched nonautistic sibling controls, which is a powerful design to control for unmeasured confounding factors shared between siblings, such as shared genetic and familial socioeconomic factors.^34^ It was clear that individuals with ASD had a greater risk of depression than their nonautistic siblings, suggesting a mechanism other than shared familial characteristics. This finding gives further credence to results among a cohort in the United Kingdom indicating that bullying in adolescence substantially mediated the association between social communication difficulties in childhood and depression at age 18 years, an association that was not confounded by polygenic risk for autism.^35^

We found that the risk of depression in ASD varied by co-occurring intellectual disability. This resonates with results of smaller studies and case reports suggesting that those with greater cognitive ability and thus insights into being different may be more prone to depression.^9,16,17,18^ While this finding may suggest an important distinction between these ASD subgroups, the weaker estimates for depression in people with ASD who have intellectual disability may also be an artifact of diagnostic overshadowing,^36^ which refers to clinicians missing treatable comorbidities in people with intellectual disability and instead attributing them to be a core feature of their intellectual disability. Individuals with lower cognitive ability may also not have sufficient verbal skills to express their difficulties and often require a diagnosis to be based on behavioral features, which may be more easily missed than verbal reports of depressive features. Future work comparing the risks of depression in individuals with ASD who also have intellectual disability vs in individuals who have intellectual disability but no ASD may provide further insights into the role of cognitive disabilities in the associations observed.

Approximately half of the individuals with ASD with depression in our sample received a diagnosis of ASD after first being diagnosed as having depression. This finding should be unsurprising to clinicians working with adolescents and adults with autism and underscores the importance of acknowledging that, despite the increased awareness and recognition, many individuals with ASD, especially those without cognitive impairments, receive a delayed diagnosis, often after experiencing other psychiatric problems.^12^ Our sensitivity analysis that excluded those who had received a diagnosis of ASD after being diagnosed as having depression found more than a 2-fold risk of depression with ASD. Although not trivial, this increased risk was lower than the risk of depression in the full sample, in which we did not specify this temporal order. In our clinical experience, individuals receiving a diagnosis of ASD later in life often report long-standing stress in relation to social isolation, bullying, exclusion, and the knowledge that they are different but without the explanatory framework of ASD to help them contextualize their difficulties. Although more research will be required to gain a fuller understanding, our results may suggest that receiving a diagnosis of ASD could partially buffer against an even greater risk of depression by helping individuals understand their difficulties and seek relevant support from educational, health, or social services. Furthermore, our sensitivity analysis accounting for childhood depression confirmed that the association between ASD and adult depression was not entirely explained by having been diagnosed as having depression during childhood and adolescence.

### Strengths and Limitations

As a total population study with a comprehensive multisource ascertainment of ASD, the study design herein reduces the possibility of selection bias and exposure misclassification. The outcome data were prospectively recorded and rely on depression diagnoses made by clinicians, thus reducing the possibility of recall and reporting bias. Because most of the Swedish population uses public services, we are likely to have captured the majority of clinically recorded diagnoses of depression. However, we have no information on undiagnosed depression in the population or on the severity or functional impact of the depressive symptoms. Misclassification because of individuals with undiagnosed depression in the cohort is likely to lead to an underestimate of the true association between ASD and depression if nondifferential in relation to our exposure or if depressed individuals with ASD are less likely to seek or engage with services to obtain a depression diagnosis. However, if the opposite is true (ie, if people with ASD are more likely than the general population to seek help for depression or are more likely than the general population to be diagnosed as having depression because of greater contact with health or mental health services), then our results may overestimate the true associations. An overestimate of true associations may also occur if people with ASD, because of greater functional impacts owing to their ASD and other comorbidities, are more likely to receive a depression diagnosis at lower levels of depressive symptoms than the general population. Other possibilities of outcome misclassification should also be considered. Making a clinical diagnosis of depression in ASD can also be complex because of atypical presentations and the need to rely on family informants.^13^ Such potential misclassification of depression by clinicians would be common to any similar study using record linkage methods, and longitudinal studies using interview or questionnaire-based methods may provide complementary evidence.^35^

## Conclusions

Findings of this work have several implications for future research and clinical practice. While our study fills a void of longitudinal studies on this topic, future research following up individuals to later in adulthood will be required to fully capture the associations between autism and depression from a life-course perspective. Further understanding of the phenomenology and measurement of depression in individuals with autism is also essential for a more nuanced knowledge of the associations we have reported. The sibling comparisons suggest the possibility of environmental pathways in the association between autism and depression, and studies identifying such pathways could assist in the development of preventive strategies or interventions. Furthermore, there is still a paucity of literature on the effectiveness of psychological^37^ or pharmacological^38^ management of depression in people with autism. Adult autism services in the West, particularly those for higher-functioning individuals without intellectual disability, are often commissioned to identify and diagnose autism rather than manage co-occurring difficulties. Because of the likelihood of a substantial overrepresentation of depression among individuals with ASD, a greater focus on timely identification and management of depression is important considering that it is a potentially treatable cause of distress, disability, and suicidal behaviors.

## Notes

Supplement. **eTable 1.** Associations Between ASD and Adult Depression Specifying That an ASD Diagnosis Must Have Preceded a Diagnosis of Depression **eTable 2.** Prevalence of Depression Diagnosed Before or After the Age of 18 Among Those Diagnosed With Autism Spectrum Disorder (ASD) With or Without Intellectual Disability (ID) **eTable 3.** Associations Between ASD and Adult Depression, Taking Into Account Depression in Adolescence
