# Supplementary material for: Association Between Autism Spectrum Disorders With or Without Intellectual Disability and Depression in Young Adulthood
Source: JAMA Netw Open. 2018 Aug 31;1(4):e181465. doi: 10.1001/jamanetworkopen.2018.1465 (PMC6324523; doi:10.1001/jamanetworkopen.2018.1465)
Supplement: Supplement. — eTable 1. Associations Between ASD and Adult Depression Specifying That an ASD Diagnosis Must Have Preceded a Diagnosis of Depression eTable 2. Prevalence of Depression Diagnosed Before or After the Age of 18 Among Those Diagnosed With Autism Spectrum Disorder (ASD) With or Without Intellectual Disability (ID) eTable 3. Associations Between ASD and Adult Depression, Taking Into Account Depression in Adolescence [file jamanetwopen-1-e181465-s001.pdf]

Supplementary Online Content

Rai D, Heuvelman H, Dalman C, et al. Association between autism spectrum disorders with or without intellectual disability and depression in young adulthood. *JAMA Netw Open*. 2018;1(4):e181465. doi:10.1001/jamanetworkopen.2018.1465

eTable 1. Associations Between ASD and Adult Depression Specifying That an ASD Diagnosis Must Have Preceded a Diagnosis of Depression

eTable 2. Prevalence of Depression Diagnosed Before or After the Age of 18 Among Those Diagnosed With Autism Spectrum Disorder (ASD) With or Without Intellectual Disability (ID)

eTable 3. Associations Between ASD and Adult Depression, Taking Into Account Depression in Adolescence

This supplementary material has been provided by the authors to give readers additional information about their work.

© 2018 Rai D et al. *JAMA Network Open*.

eTable 1. Associations Between ASD and Adult Depression Specifying That an ASD Diagnosis Must Have Preceded a Diagnosis of Depression

	Cases versus population controls	
Autism Spectrum Disorder (ASD)	OR (95% CI)<br>Model 1	OR (95% CI)<br>Model 2
All ASD	2.61 (2.38 – 2.86)<br>p<0.001	2.26 (2.07 – 2.48)<br>P<0.001
ASD without intellectual disability (ID)	3.04 (2.75 – 3.37)<br>P<0.001	2.57 (2.32 – 2.85)<br>P<0.001
ASD with ID	1.68 (1.37 - 2.05)<br>P<0.001	1.54 (1.26 – 1.89)<br>P<0.001

Notes: (1) Modified Poisson regression with cluster robust standard errors. (2) Model 1 adjusted for age and sex. (3) Model 2 adjusted for age, sex, maternal and paternal age, parental educational attainment, family disposable income quintile, foreign birth of child or parents, and maternal and paternal psychiatric history.

© 2018 Rai D et al. *JAMA Network Open*.

eTable 2. Prevalence of Depression Diagnosed Before or After the Age of 18 Among Those Diagnosed With Autism Spectrum Disorder (ASD) With or Without Intellectual Disability (ID)

	Diagnosis of depression			
Autism Spectrum Disorder (ASD)	None	Diagnosis before age 18 years	Diagnosis of depression after age 18 years	Diagnosis of depression at any time
	% (n)	% (n)	% (n)	% (n)
No ASD	93.1 (204,549)	1.5 (3,251)	6.0 (13,114)	6.9 (15,220)
All ASD	75.8 (3,087)	7.4 (303)	19.8 (808)	24.2 (986)
ASD without intellectual disability (ID)	70.6 (2,067)	9.3 (272)	24.1 (704)	29.4 (860)
ASD with ID	89.0 (1,020)	2.7 (31)	9.1 (104)	11.0 (126)

© 2018 Rai D et al. *JAMA Network Open*.

eTable 3. Associations Between ASD and Adult Depression, Taking Into Account Depression in Adolescence

	Cases versus population controls		
Autism spectrum disorder (ASD)	OR (95% CI)<br>Model 1	OR (95% CI)<br>Model 2	OR (95% CI)<br>Model 3
All ASD	4.19 (3.94 - 4.47)<br>P<0.001	3.64 (3.41 - 3.88)<br>P<0.001	2.92 (2.72 - 3.13)<br>P<0.001
ASD without intellectual disability (ID)	5.05 (4.73 - 5.39)<br>P<0.001	4.28 (4.00 - 4.58)<br>P<0.001	3.27 (3.03 – 3.53)<br>P<0.001
ASD with ID	1.96 (1.63 - 2.35)<br>P<0.001	1.81 (1.51 - 2.17)<br>P<0.001	1.68 (1.40 - 2.02)<br>P<0.001

Notes: (1) Modified Poisson regression with cluster robust standard errors. (2) Model 1 adjusted for age and sex. (3) Model 2 adjusted for age, sex, maternal and paternal age, parental educational attainment, family disposable income quintile, foreign birth of child or parents, and maternal and paternal psychiatric history. (4) Model 3 adjusted for all prior covariates and depression before the age of 18.

© 2018 Rai D et al. *JAMA Network Open*.
